# Wireless Manipulation Mechanism and Analysis for Actively Assistive Pinch Movements

**DOI:** 10.3390/s21186216

**Published:** 2021-09-16

**Authors:** Dong-Min Ji, Won-Suk Jung, Sung-Hoon Kim

**Affiliations:** 1 Department of Electronics Convergence Engineering, Wonkwang University, 460 Iksandae-ro, Iksan 54538, Korea; anggole012@wku.ac.kr; 2 School of Mechanical Engineering, Chungnam National University, Daejeon 34134, Korea; 3 Wonkwang Institute of Materials Science and Technology, Wonkwang University, 460 Iksandae-ro, Iksan 54538, Korea

**Keywords:** electromagnetic manipulation system, magnetic actuation, multilink magnetic assistive device, pinch motion, wireless finger rehabilitation

## Abstract

Pinching motions are important for holding and retaining objects with precision. Therefore, training exercises for the thumb and index finger are extremely important in the field of hand rehabilitation. Considering the need for training convenience, we developed a device and a driving system to assist pinching motions actively via a lightweight, simple, and wireless mechanism driven by the magnetic forces and torques generated by magnets attached to the tip of these two fingers. This device provides accurate pinching motions through the linking structures connecting the two magnets. The fabricated device has minimal mechanical elements with an ultralightweight of 57.2 g. The magnetic field, the intensity of which is based on the time variant, generates a pinching motion between the thumb and index finger, thus rendering it possible to achieve repetitive training. To verify the generation of an active pinching motion, we fabricated a finger model using a 3D printer and a rubber sheet and observed the active motions generated by the newly developed device. We also verified the performance of the proposed mechanism and driving method via various experiments and magnetic simulations. The proposed mechanism represents an important breakthrough for patients requiring hand rehabilitation and wearable assistive motion devices.

## 1. Introduction

Hand function and usability play an important role in determining the quality of human life. Hand injuries and the inability to use one’s hand because of a stroke are rapidly increasing [1]. Therefore, to restore hand functions, post-stroke care and recovery are very important [2,3,4]. In general, post-stroke care can be administered in the form of rehabilitation. Various devices and methods have been studied for hand rehabilitation purposes. In many cases, rehabilitation measures involve utilizing the patient’s own hand strength and assistive training using external devices. In the former, the patients are encouraged to train by themselves without additional assistance from devices. Rehabilitation devices can be categorized into passive and active types depending on whether an external force is generated. In the case of the passive types, the patients are trained and rehabilitated using assistive content such as virtual reality or games or both without additional external force. The passive types of assistive devices involve the wearing of gloves composed of various position sensors to check hand conditions [5,6,7,8,9]. However, the passive types have the disadvantage that they are impossible to use for training without the patient’s hand force. Therefore, in the early state of rehabilitation, such a device requires the generation of an active force for actively assisting training.

The active types, which can be further categorized into hard and soft types according to their body materials, can support hand movement by generating force. The hard-type devices are made of a rigid body material and do not deform. An exoskeleton-type device exemplifies the mechanism of the hard type. It is generally controlled by an electric motor and wire, and it also applies pneumatic and hydraulic pressure. In the case of the exoskeleton type, it can generate a large force of 10 to 50 N, but it is structurally complex, bulky, difficult to wear, and has a weight of approximately 100 to 500 g. Furthermore, high precision control is required because a large force is applied to the finger [10,11,12,13,14,15,16].

The soft-type devices are made of an elastic material and are flexible. In general, soft actuators are controlled using pneumatic or hydraulic pressure [17,18]. Soft-type devices are lightweight, with weight typically in the range of 100 to 300 g. Furthermore, the devices have a relatively simple structure, which improves on the disadvantages of the hard-type devices. However, the soft-type devices require tubes on the hardware for motion control, which can cause the patient to feel discomfort [19,20,21,22]. The soft-type devices can also generate strong forces of up to 20 N. Most of the robotic hand-type rehabilitation devices primarily provide four-finger movement that excludes the thumb. However, among the various functions performed by the human hand, thumb functions are very important because they provide more than 40% of the total functionality for the human hand [23]; therefore, thumb rehabilitation is necessary in the event of an injury [24,25,26]. The pinch motion plays an important role in hand functions, especially those involving pinching and holding objects with precision using the thumb and index finger. In particular, repetitive pinching motions are considered important in thumb rehabilitation training. Therefore, rehabilitation devices to assist pinch motions are being actively developed.

Guo et al. [27] developed an exoskeleton finger rehabilitation robot driven by the direct coupling of a hard-type exoskeleton device and a dc motor. The device is capable of generating a strong torque of 23.5 Nm and weighs 136 g. The weight of the exoskeleton was subsequently reduced to 52 g by disconnecting the dc motor and powering it through a wire. Furthermore, a thumb structure was also added [28]. Agarwal et al. [29] developed an index finger-based hard-type device. Their device is capable of precise torque control using a dc motor, compression spring, and Bowden cable, and it is lightweight at 80 g. A thumb-based hard-type device was subsequently developed. This hard-type device is capable of generating a maximum torque of 0.3 Nm and weighs 136 g [30].

Cempini et al. [31] developed HandeXos (HX)—a hard-type rehabilitation device. HX has a self-aligning joint mechanism that changes the joint position of the robot according to the finger movement for natural finger movement. Furthermore, it is manufactured in a modular format to provide a comfortable fit, and the total weight is 438 g. Marconi et al. [32] developed an improved rehabilitation device, called HandeXos-beta (HX-β), to enable a more natural pinch motion by adding a rotation axis to the CMC joint of HX. In addition, HX-β uses an elastic actuator for precise torque control and generates a driving force of approximately 4N at the fingertip. Gabardi et al. [33] developed an exoskeleton device that can support thumb movement. The structure, which is manufactured by directly coupling a linear motor, is composed of 12 rotating parts, facilitating natural movement. The device generates a gripping force of up to 3.11 N. The robotic hands outlined above have both advantages and disadvantages. The disadvantages include having a complex structure, large volume, and difficulty for the wearer owing to the frame being made of a rigid body, with components such as a motor, wire, and complex links.

This paper proposes a mechanism that actively assists the pinch motion using magnetic actuation without wires and electrical motors. Specifically, only magnets and magnetic field manipulations via magnetic torques and forces are employed. From our preliminary research, we verified that the finger motions can be controlled with magnetic torques and forces and minimal mechanization [34,35]. The proposed mechanism and driving method assist pinching motions via joint two-link structures that include magnets placed on the tip of the thumb and index fingers and directed torque control using an alternating magnetic field. The two-link mechanism allows stable pinch motions without interference between the attached magnets. The only attachment on the hand is the two-link structure containing the magnets, which is a very lightweight at only 57.2 g. To verify the active movements via magnetic actuation, the driving method was evaluated via electromagnetic simulations. Furthermore, to confirm that the device is actively operated via magnetic actuation, it was installed on a finger structure, and tests and motion analyses were performed. Thereafter, it was demonstrated that pinching motions could be achieved via magnetic actuation in real-world applications by mounting the device on the hand of a human subject.

## 2. Strategies for Pinch Motion Training

### 2.1. Magnetic Operation and Manipulation Principle

We propose a new strategy for active assistance pinch-motion training using magnetic actuation. Our goals are to achieve wireless operation as well as a simple and lightweight structure to provide training convenience. The joints of the thumb and index finger must be able to rotate in order to support active pinching motions with minimal external mechanisms. In particular, the position of the fingertips is represented by the summation of the rotation angles of each finger joint. Therefore, the magnetic mechanisms are installed on the fingertips to create natural pinch motions.

**Figure 1:**
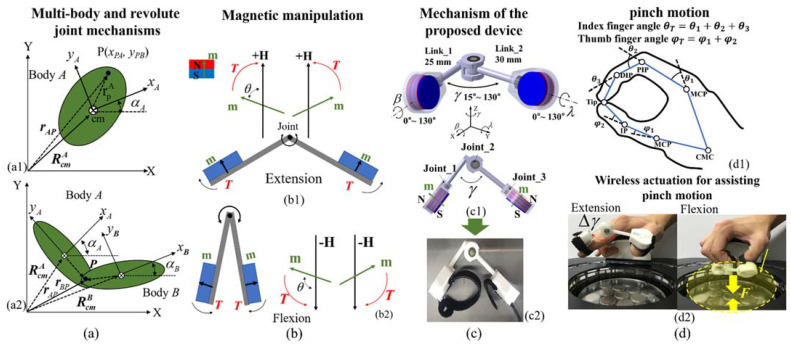
Principles involved in wireless finger rehabilitation: (**a**) free body diagram (FBD) of multibody and revolute joint mechanisms; (**b**) magnetic actuation method using the multibody mechanism for pinching motions (extension and flexion); (**c**) configuration and mechanism of the proposed device; (**d**) pinch motion with fingertip positions and actual pinching movement using the developed device.

Figure 1 shows our proposed strategy for supporting pinch motions using magnetic actuation with manipulation. We considered the basic concepts of the parallel two-link mechanism comprising permanent magnets. Links 1 and 2 must move in opposite directions because the pinch motion moves the thumb and index finger in opposite directions. The links are parallel structures and produce independent movements. Thus, each link can be analyzed as a one-link manipulator. To produce movements in opposite directions between the two links in the applied magnetic field, we mounted permanent magnets on the links, and the directions of the magnetic moments (***m***) are the same (parallel) as those of movement, as shown in Figure 1b. The role of links 1 and 2 is to maintain a stable pinching motion. Note that if only the magnets are attached to the fingertips without the links, the magnets may stick together in the final pinch motion, causing difficulty separating the fingers for the next repetition. The proposed device uses multibody and revolute joint mechanisms. The position of joint *P* on body A relative to the global coordinate system is expressed as follows:

(1) $$R_{p}^{A}=R_{cm}^{A}+r_{p}^{A}=R_{cm}^{A}+r_{A}r_{p}^{A},$$

where $R_{cm}^{A}$ is the position vector of the origin of the *x_A_* and *y_A_* coordinate system with respect to the global XY coordinate system, and $r_{p}^{A}$ is the position vector of point *P* on body *A* with respect to the *x_A_* and *y_A_* coordinate system. $r_{A}$, which is the transformation matrix from the XY to the *x_A_* and *y_A_* coordinate systems, and $r_{p}^{A}$ are given by (2) and (3), respectively:

(2) $$r_{A}=\left(\begin{array}{l} \cos\alpha_{A}-\sin\alpha_{A}\\ \sin\alpha_{A}\cos\alpha_{A} \end{array}\right),$$

(3) $$r_{P}^{A}=\left(\begin{array}{l} x_{PA}\\ y_{PA} \end{array}\right),$$

where $\alpha_{A}$ is the angle that is relatively oriented to the global XY coordinate system. Next, we considered the equation of motion for the revolute joint. This joint connects two bodies *A* and *B*, as shown in Figure 1(a2). Bodies *A* and *B* have the same translational motion as the joint but can have relative rotations. Therefore, the equation of motion for the revolute joint and the two bodies can be expressed as follows:

(4) $$\begin{array}{ccc} r_{AP} & = & r_{BP}\\ R_{cm}^{A}+r_{A}r_{p}^{A}-R_{cm}^{B}-r_{B}r_{p}^{B} & = & 0 \end{array}$$

where $r_{AP}$ is the position vector of point P on body A with respect to the global XY coordinate system, $r_{BP},R_{cm}^{B},\;r_{B}r_{p}^{B}$ are the position vectors with respect to body *B*. Equation (4) can be rewritten as (5):

(5) $$\left\{\begin{array}{l} 0\\ 0 \end{array}\right\}=\left\{\begin{array}{l} X_{cm}^{A}\\ Y_{cm}^{A} \end{array}\right\}+[\begin{array}{l} \cos\alpha_{A}\\ \sin\alpha_{A} \end{array}\begin{array}{l} -\sin\alpha_{A}\\ \cos\alpha_{A} \end{array}]\left\{\begin{array}{l} x_{p}^{A}\\ y_{p}^{A} \end{array}\right\}-\left\{\begin{array}{l} X_{cm}^{B}\\ Y_{cm}^{B} \end{array}\right\}-[\begin{array}{l} \cos\alpha_{B}\\ \sin\alpha_{B} \end{array}\begin{array}{l} -\sin\alpha_{B}\\ \cos\alpha_{B} \end{array}]\left\{\begin{array}{l} x_{p}^{B}\\ y_{p}^{B} \end{array}\right\}.$$

Using the revolute joint mechanism, we connected the two links to the joint and installed a permanent magnet on each link. The two links are rotated in the applied magnetic field because of the magnetic torque, as shown in Figure 1b. The polarity of the permanent magnet installed on each link is oriented in the same direction to ensure that the movements of the thumb and index finger can be reversed, as in the pinch motions performed with these fingers. Therefore, the two links rotate in opposite directions under the same magnetic field conditions. Figure 1(b1) shows the extension motion when the applied magnetic field is positive (+H); however, a negative field (−H) generates flexion motion because of the magnetic torque direction (see Figure 1(b2)). When an external magnetic field exists, the magnets align in the direction of that field; thus, two motions, extension and flexion, can be created with the proposed mechanism and by controlling the magnetic field.

**Figure 2:**
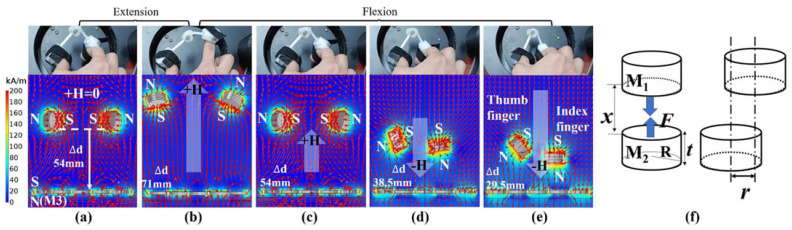
Magnetic simulation results according to changes in finger position due to driving current variations: (**a**,**b**) initial position and maximum extension of fingers during 0 to 25 A; (**c**–**e**) variations in flexion motions up to –25 A and their magnetic couplings in magnetic simulations; (**f**) magnetic force between two permanent magnets. (see Video S1).

The magnetic torque between a permanent magnet and a magnetic field is expressed as follows:

(6) $$T=\mu_{0}m\times H=\mu_{0}mH\sin\theta$$

where ***T*** is the magnetic torque, ***m*** is the magnetic moment of the permanent magnet, ***H*** is the magnetic field strength, $\mu_{0}$ is the vacuum permeability, and θ is the angle between the applied field and the magnetic moment direction. Figure 1(d1) shows the pinch motion and joint angles for the thumb and index finger. The index finger has three joints: distal interphalangeal (DIP), proximal interphalangeal (PIP), and metacarpophalangeal (MCP). The thumb has two joints: interphalangeal (IP) and MCP. The pinch motion occurs when the thumb and index fingertips meet. Under these conditions, the total angles of the thumb and index fingertips are $\theta_{T}=\theta_{1}+\theta_{2}+\theta_{3}$ and $\varphi_{T}=\varphi_{1}+\varphi_{2}$, respectively. To assist pinching movements actively, we attach the proposed device to the fingertips and change the direction of the magnetic torques. Under these movements, at the completion of the final pinch motion, the magnetic force between the magnets is generated, as shown in Figure 2f; this magnetic force is expressed as follows [35]:

(7) $$F\approx -\frac{1}{4}\pi M_{s}^{2}R^{4}\sum_{ij=0}^{1}\frac{{\left(-1\right)}^{i+j}}{{\left(x+it_{1}+jt_{2}\right)}^{2}}\left[1-\frac{3}{2}\frac{r^{2}}{{\left(x+it_{1}+jt_{2}\right)}^{2}}\right],$$

where *M_s_* is the magnetization and *R* and *x* are the magnet radius and distance between magnets, respectively. *t* is the thickness of the magnet, and *r* is the lateral displacement. When the thickness of the magnets is the same and the lateral displacement is zero, ***F*** can be rewritten as follows:

(8) $$F\approx -\frac{1}{4}\pi M_{s}^{2}R^{4}\left[\frac{1}{x^{2}}+\frac{1}{{\left(x+2t\right)}^{2}}-\frac{2}{{\left(x+t\right)}^{2}}\right].$$

Figure 2b,c show the magnetic actuation for extension between the thumb and index finger at the applied currents of 25 and 7 A; extension occurs because of the positive magnetic torque (see Figure 1(b1)).

### 2.2. Fabrication and Configuration of the Magnetic Finger Device with Magnetic Actuation

The proposed device consists of two magnet containers, two links, and three joints (four bearings), as shown in Figure 1c. Here, joint 2 comprises two bearings and facilitates the extension and flexion motions between the thumb and index finger. When the device is mounted on the fingertips, changes in the angles of the fingertips cause natural tip-pinch motions because the position of the fingertips is determined by the sum of their joint angles (see Figure 1(d1)). Joints 1 and 3 (two bearings) provide the rotation of the magnet containers, and the rotation of the containers provides convenience when attaching the device to the fingers. When the final pinch motion is reached, the rotation of the magnet containers can naturally support a twisting motion because the actual fingertips perform these types of small motions. Joints 1 and 3 produces the rotation angles β and λ up to 130°. The interlink angle **γ** between links 1 and 2 varies from a minimum of 15° to a maximum of 130°. The inner diameter and height of the container are 20.4 and 11 mm, respectively. Thus, we installed three disc-type permanent magnets with the same diameter and thickness of 20 and 3 mm, respectively. The type of magnet used was N27, and its magnetic remanence Br was 1.07 T. Figure 1(c2) shows the fabricated device containing the disc-type magnets. As described before, the fabricated device actively supports pinch motions via the generated magnetic torques and forces. Thus, we investigated the relationship between the driving magnets and the magnetic field through magnetic simulation, as shown in Figure 2. The simulations were performed under the same conditions as the actual pinch motion experiments. M1 and M2 are the magnets in the device, and M3 is the magnet array in the driving coil. In the newly developed system, the initial position of the device is at a distance of 54 mm from M3, and the magnets (M1 and M2) of the finger device produce repulsive forces with an angle **γ** of 74° when the applied magnetic field is zero, as shown in Figure 2a.

### 2.3. Control System Configuration and Instrumentation of the Driving Magnetic Field

**Figure 3:**
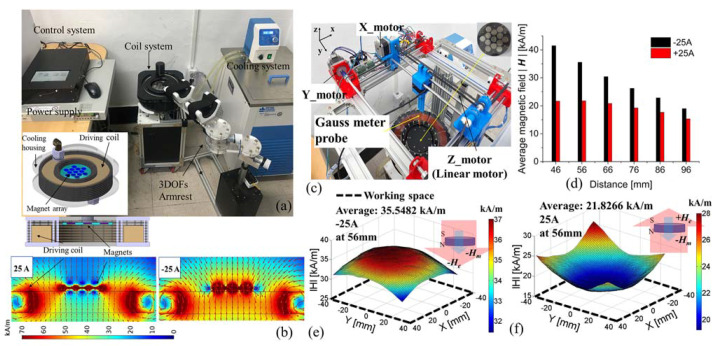
Control system analysis using magnetic simulation and magnetic field measurement: (**a**) control system configuration; (**b**) simulation results including M3 at 25 and −25 A; (**c**) developed three-axis measurement system and LabView-based instrumentation software; (**d**) measured average magnetic field strength with −25 and 25 A driving currents according to changes in height up to 96 mm; (**e**,**f**) field distributions for −25 and 25 A, respectively, at 56 mm height: H_e_ and H_m_ are the magnetic fields of the electromagnets and M3 (permanent magnets), respectively. (see Video S2).

Figure 3a shows the overall control system configuration. The system consists of the driving coil, three-axis armrest, dc power supply, control circuit, and cooling system. To generate a bipolar signal, we designed a bipolar circuit in which a solid-state relay is incorporated into the control circuit. The control circuit with dc power supply provides a current of up to ±25 A to the driving coil, which generates heat exceeding 100 °C on the coil. When the cooling system is set to achieve a cooling temperature of 5 °C, a constant driving-coil temperature guarantees a constant current supply and magnetic field, which are very important for the generation of continuous changes in the magnetic field intensities in the workspace; hence, finger flexion and extension are achieved. A LabView-based control program was developed for real-time observation of the driving coil and for hand motion analysis. For wireless finger rehabilitation, we developed an electromagnetic control system comprising a driving coil and a permanent magnet array (M3) to generate the driving magnetic force. The driving coil produces the main magnetic fields for finger flexion and extension (i.e., pinch motions).

We investigated the combined magnetic fields between the magnet array and the coil; here, the driving coil was fabricated using 370 turns of 2-mm-thick wire with inner and outer diameters of 180 and 260 mm, respectively. The fabricated magnet array was a disk-shaped permanent magnet with 3 mm thickness and 10 mm diameter, and it was composed of 13 magnets in total with the upper side being the S pole. M3 was positioned in the inner space of the driving coil. Figure 3b shows the results of a COMSOL magnetic distribution simulation of the combined magnetic field from the magnets with the coil at the driving currents of 25 and –25 A. When a 25 A current is applied, the coil becomes an electromagnet with the top and bottom as the N and S poles, respectively. Thus, the electromagnet polarity is opposite to that of M3, and there is a very-low-strength magnetic field in the M3 region. However, the overall magnetic field distribution is directed upward from the coil, causing finger flexion. When a −25 A current is applied, the polarity directions are the same for both the electromagnet and the magnet array. In this case, the flux is directed from the top to the bottom of the coil, and the field direction causes finger extension. Through the magnetic simulation performed during this study, we were able to predict the field distributions generated in the workspace by the coil and magnet array. In conjunction with the simulation, we measured the actual magnetic field distributions using a gauss meter (F. W. Bell 5180 Milwaukie, OR, USA).

Note that we developed a tailored measurement system involving both hardware and software, as shown in Figure 3c. The employed hardware was a three-axis auto-scanning machine, in which the measurement probe could be moved with a minimum measurement distance of 1 mm. In these measurements, the XY plane was measured at 5 mm intervals, and the height (Z-axis) was measured at 10 mm intervals from the surface up to a maximum distance of 100 mm. The measured data were transformed into a three-dimensional magnetic field distribution in the measurement program. In this system, the hand is initially positioned at a height of 56 mm from the coil, because this corresponds to the height of a hand at rest. Therefore, the highest possible finger trajectory point was 100 mm. Figure 3d shows the average magnetic field strengths for each height observed at intervals of 10 mm from a height of 46 mm to a maximum of 96 mm at the applied currents of −25 and 25 A. When the average magnetic field is measured for each height by applying a current of −25 A, the decreased rate is large according to the height change because it is in the same direction as the field of the M3 array. On the other hand, the direction of the magnetic field for the current of 25 A is opposite to that of the M3 array. Therefore, the rate of change of the magnetic field is low according to the height change because of the cancellation of the magnetic field. Flexion between the thumb and index finger occurs when the driving current changes from 25 to −25 A, and extension occurs when the current changes from –25 to 25A, resulting in a pinch motion. The maximum flexion motion occurs between 46 and 56 mm, and the maximum extension motion is achieved at approximately 96 mm. Figure 3e,f shows the magnetic field distributions in the working space at a height of 56 mm and for applied currents of −25 and 25 A, respectively, which in turn produced average fields of 35.548 and 26.826 kA/m, respectively.

## 3. Experimental Results and Analysis

For the experimental verification of the actively assisted pinch motion device based on the proposed mechanism and control method, we performed two types of experiments.

First, we fabricated a passive robotic finger and applied the developed device to verify that the pinch motion was actively generated by magnetic actuation. Second, we attached the device to the fingers of a human subject and observed the pinch motion.

### 3.1. Observation of the Robotic Finger Pinch Motion by Magnetic Actuation

**Figure 4:**
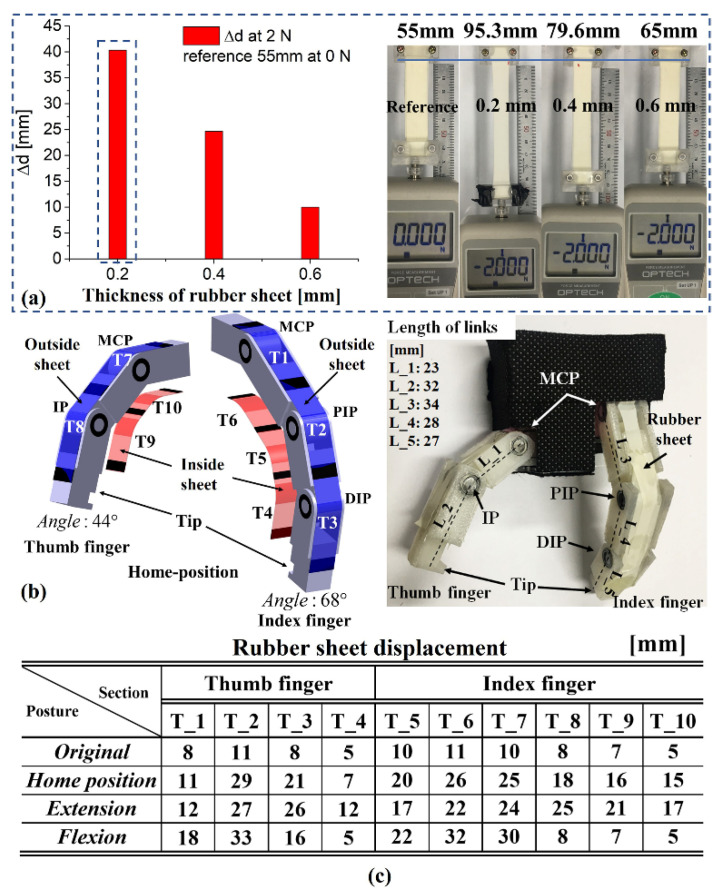
Basic properties of the rubber sheet: (**a**) displacements of the rubber sheet according to the sheet thickness at the applied force of 2 N. (**b**) Robotic finger structure and configuration. (**c**) Fabricated robotic finger: length of thin rubber pad and displacements at each posture (condition).

To verify active pinch motions using magnetic actuation, we prepared two passive-type robotic fingers. The robotic fingers mimic the structures of the human thumb and index finger. In particular, the attached natural rubber sheets maintain the posture of the fingers to act as artificial tendons. To employ the rubber sheets with the robotic fingers, we examined the increased displacement by applying a 2N force to the rubber sheets with constant width and length of 9 and 55 mm, respectively, and varying thicknesses of 0.2, 0.4, and 0.6 mm, as shown in Figure 4a. Under these three thickness conditions, each rubber sheet produced the displacements of 40.3, 24.6, and 10 mm, respectively. Figure 4b shows the configuration of the robotic fingers and the structure of the rubber sheets. T1 to T10 are the rubber sheets in the configuration. Figure 4c shows the fabricated robotic fingers and the displacements of the rubber sheets to mimic the initial posture and movement of a human thumb and index finger.

The thumb consists of two links and four rubber sheets (T7 to T10), and the index finger consists of three links and six rubber sheets (T1 to T6). For use as artificial tendons, the applied natural rubber sheet thickness was 0.2 mm. We adjusted the length of the rubber sheet connecting each link to create the initial posture of a human finger. To generate the home position, the total length of the inside rubber sheet is shorter than that of the outside rubber sheet.

**Figure 5:**
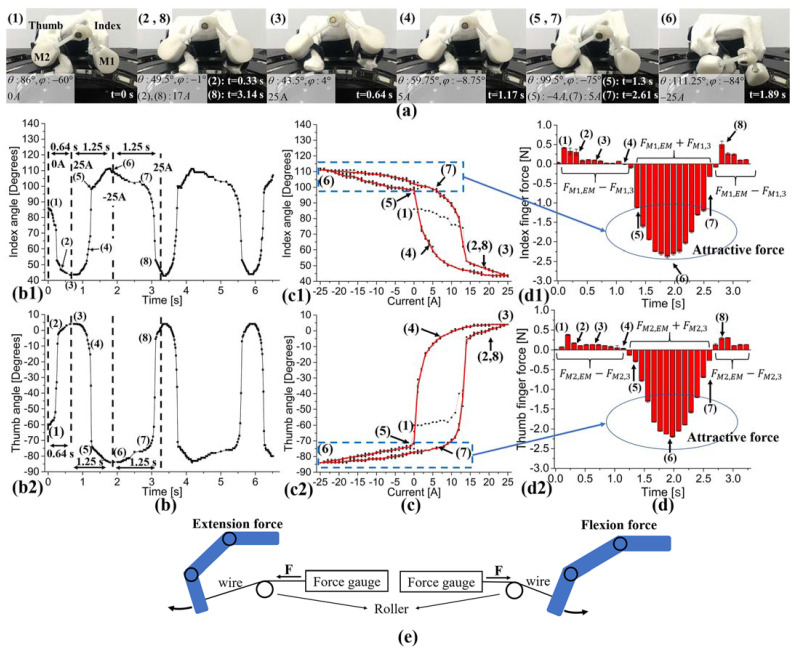
(**a**) Thumb and index finger postures using the robotic finger during pinch motions and their magnetic coupling. (**b**) Thumb and index finger angle change observations during pinch motions according to changes in time. (**c**) Thumb and index finger angle change observations during pinch motions according to changes in driving current. (**d**) Thumb and index finger force variation observations during pinch motions. (**e**) Finger force measurement method during the pinch motion. Flexion (3–6) occurs for 25 to −25 A and extension (6–8 and 3) occurs for –25 to 25 A. (see Videos S3 and S4).

Using the fabricated passive robotic finger, we investigate active pinch motions by magnetic actuation through experiments and magnetic simulation, as shown in Figure 5. To generate active pinch motions using the device, a current in the range of ±25 A was applied at 0.4 Hz; then, we observed the variations in pinch motions through a video camera. For this analysis, we first performed experiments to obtain actual finger orientations corresponding to the driving currents. We defined the fingertip angles θ and φ as 0° when the fingers were fully extended. Therefore, when the fingers generate extension, the angles decrease from the initial angles; whereas when flexion occurs, the angles increase (see Figure 1(d1)). The numbers (1) to (8) in Figure 5 indicate the results under the same conditions. The magnet of the finger device is arranged in the direction of the applied magnetic field because of magnetic torque. The home positions of the two fingers (index θ and thumb φ) are 86° and –60°, respectively, for the applied current of 0 A (H = 0). Therefore, for an increasing positive current, increasing the magnetic torque extends the finger. For 25 A, the thumb and index finger reached a maximum angular extension displacement of up to 42.5° ($\theta_{T}=43.5^{\circ }$) and −64° ($\varphi_{T}{=4}^{\circ }$), respectively, from the reference angles, as shown in Figure 5a (3). On the other hand, for increasing negative current, the torque direction becomes opposite and flexion occurs, a maximum angular displacement of the index and thumb showed 25.25° ($\theta_{T}=111.25^{\circ }$) and –24° ($\varphi_{T}{=-84}^{\circ }$), respectively, from the reference angles, as shown in Figure 5a (6).

In particular, an attractive force is generated between the magnets of the devices and the magnet array M3, and the coupling force is maximized at the applied current of −25 A (see Figure 5a (6)), which can be confirmed through the simulation.

Figure 5b,c show variations in finger motion according to changes in time and driving current, respectively. In particular, the cause of these variations during the pinch motion can be confirmed through force variations observed in Figure 5d. Figure 5b,d show variations in the angles of the thumb and index and in force according to changes in time, respectively. Since the thumb and index finger are moving in opposite directions, the movement direction is reversed at the same time for both fingers. As a result of the opposite movement direction of the two fingers, the thumb and index finger angles are indicated as minus and plus, respectively, as shown in Figure 5(b1,c1) (index finger) and Figure 5(b2,c2) (thumb finger). Although the movement starts at 0 A (1), the repetitive pinch movement repeats in two sections between 25 and −25 A ((3) to (6): flexion) and –25 and 25 A ((6), (8), and (3): extension). In Figure 5, the section from (3) to (4) slowly generates a flexion motion (index finger: 43.5° to 59.75° and thumb finger: 4° to –8.75°) because the force does not work concurrently with the magnet array (M3); whereas in the section from (4) to (5), the flexion motion proceeds rapidly by the magnetic coupling force between the device magnets (M1 and M2) and the magnet array (M3) (index finger: 59.75° to 99.5°; thumb finger: –8.75° to –75°).

In addition, the coupling force maintains a pinching state and causes a low rate of change of the finger angle as in position (5) to (7): the extension motion shows a large rate of change from approximately 10 A. When extension occurs, the two fingers gradually move away from the driving coil, and the magnetic field strength and driving force are reduced. Therefore, the angle rate of change decreases from approximately 15 to 25 A in the motion characteristics of the finger, as shown in Figure 5(c1,c2). When analyzing the movement or angle of a finger with time, it is very difficult to analyze the intrinsic motion characteristics of the finger in repetitive motions. Therefore, we analyzed the angular change of the finger according to the changes in the driving current, and we observed the finger trajectory during pinch motion, as shown in Figure 5c. The black dots represent the trajectory for three cycles, and the red line is the average value of three cycles. In our previous study, although we proposed a hysteresis analysis method of finger movement, statistical analysis of the hysteresis, motion analysis according to the time change, and comparative analysis were not performed. Therefore, this study used statistical analysis of hysteresis analysis, motion analysis according to time change, and force analysis to be compared and analyzed by the external force of the device. In particular, our recent study established a system for statistical analysis of finger motion and hysteresis characteristics using a magnetic field-driven finger exercise device [36]. We can understand the finger pinch motion tendency caused by the driving system and the finger conditions through the hysteresis characteristics. The hysteresis curve of the finger motion reflects the mechanical and material properties of the fabricated finger.

During the pinch motion, the components of the proposed control system and device generate a magnetic force. This magnetic force can be explained by dividing it into extension and flexion areas. Figure 5d shows the result of measuring the force during the pinch motion. To observe the finger forces, we connected the fingertips and the force gauges with a wire and measured extension and flexion force, as shown in Figure 5e. $F_{M1,EM}$ is the coupling force between magnet1 (M1 on the index finger) and the electromagnet (coil).

$F_{M2,EM}$ is the force between magnet2 (M2 on the thumb finger) and the electromagnet. $F_{M1,3}$ is the force between M1 and the magnet in the driving coil (M3). $F_{M2,3}$ is the force between M2 and M3. When we compare the force for the extension and flexion motions, the forces for flexion are higher. Among the forces for extension, the forces at point (3) in the thumb and index finger are 0.071 and 0.128 N, respectively, whereas the flexion forces at point (6) in the thumb and index finger are approximately –0.23 and –2.2 N, respectively. Here, minus indicates the force direction: extension is positive force and flexion is negative force. There are two reasons why the extension forces are lower than those of flexion. First, in the case of extension, the finger device moves away from the coil, reducing the influence of the magnetic field. Second, the force is generated between the finger device and the coil as well as between the finger device and the array magnet. In the case of extension, the direction of these two forces is opposite ($F_{M1,EM}-F_{M1,3}\;\mathrm{or}\;F_{M2,EM}-F_{M2,3}$), and in the case of flexion, both forces have the same direction ($F_{M1,EM}+F_{M1,3}\;\mathrm{or}\;F_{M2,EM}+F_{M2,3}$). Therefore, the flexion force is greater. In summary, we were able to quantitatively analyze the pinch motion caused by a magnetic field using a passive robotic finger.

### 3.2. Observation of Human Finger Pinch Motion by Magnetic Actuation

**Figure 6:**
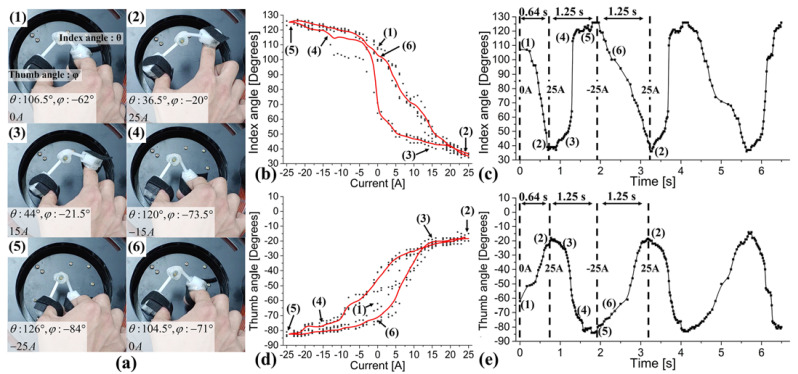
(**a**) Thumb and index finger postures during pinch motions using the proposed mechanism. (**b**) Index finger angle variations according to changes in time. (**c**) Hysteresis property of the index finger: index finger angle variations according to changes in the driving current. (**d**) Thumb angle variations according to changes in time. (**e**) Hysteresis property of the thumb: thumb angle variations according to changes in the driving current.

Next, we experimentally observed the occurrence of active pinch motions by applying the fabricated device to the hand of a healthy human subject. Depending on the condition of a person’s hand, the pinch motion results produced by the device will vary. Figure 6a (1–6) shows the postures of actual pinch motions for one cycle using the developed device. The eight postures are the common points (1) to (6) in the results shown in Figure 6b–e. Figure 6b,d is the index and thumb angle change observations for repeated pinch motions during approximately 6 s (2.5 cycles). The black dots represent the trajectory for three cycles, and the red line is the average value of three cycles. These results confirm that the human finger was also able to generate stable pinch motions using the fabricated device with magnetic field control. Since the fingers are moving in opposite directions, the angle change has a phase difference of approximately 180°. Furthermore, because the index finger is longer than the thumb—the index and thumb lengths from the MCP joint are 77 and 55 mm, respectively—the angle change of the index finger is larger in the pinch motion. In the experiment, the maximum angular displacement difference of the index and thumb was observed at approximately 90 and 64°, respectively.

Through these results, we can verify that the pinch motion can be actively generated through the proposed method. Figure 6c,e show the hysteresis properties of the index finger and thumb for extension and flexion according to changes in the driving current. These results show the motion characteristics during extension and flexion of the index finger and thumb finger when using the newly developed device with the control system. In addition, because these results reflect the anatomical and dynamic characteristics of the fingers, and each person has different characteristics, results may vary from person to person. In the results, 0 to 0.64 s is the first extension from 0 to 25 A. Therefore, we can only observe 0 to 0.64 s in the first cycle. In general, the flexion process can be observed from 25 to −25 A, whereas the extension process can be observed in changes from −25 to 25 A. This is similar to the robotic finger case.

In the case of index finger extension, a sudden change in angle does not occur, because the position of the finger gradually moves away from the coil. Since the magnetic device is mounted on the fingertip, in the case of flexion, when the position of the finger approaches the coil, the IP joint angle easily changes and a fast movement occurs owing to the magnetic force of the magnet array (see Figure 4). Rapid flexion motion was observed between 0 and –5 A, as shown in Figure 6c. Since the thumb is short between the fingertip and the IP joint, and it has an effect on both IP and MCP joints, there is no sudden change in angle compared to the PIP joint of the index finger during extension and flexion. In addition, because the direction of M2 on the fingertip causes a low magnetic coupling force ($F_{M2,3}$) between this magnet (M2) and the magnet array (M3) (see Figure 2e), the angle change pattern during the extension and flexion process is almost the same, as shown in Figure 6e.

We applied the proposed device and driving method to both a robotic hand and a human hand in order to compare and analyze the pinch motion generated by magnetic actuation. In particular, the pinch motion was compared and analyzed according to the changes in time and driving current. Through a time series of motion changes, we were able to confirm the angular change occurring during repetitive pinch motion, whereas angular changes of the pinch motion according to changes in the driving currents showed the structural characteristics of the fingers and the dynamic characteristics by the system configuration. Through hysteresis characteristics, we were able to analyze accurately the conditions of flexion and extension of two fingers during pinch motions. Therefore, the hysteresis characteristics will vary depending on the recovery abilities of the patient’s fingers. Hence, continuous observation of these dynamic characteristics could be an important way to monitor the recovery process of the patient.

## 4. Discussion and Conclusions

In this paper, we presented a newly developed device and a proposed driving method to generate pinch motions using magnetic actuation (see Video S5). A revolute joint connects the two links between magnets, and a permanent magnet is attached to each link to apply a pinch motion via magnetic torque and force. Thus, the proposed method and mechanism facilitate the fabrication of a very simple and lightweight (57.2 g) structure for a simple, easy to wear, and convenient device.

To generate a pinch motion, a magnetic field is generated in the current range of −25 to 25 A. When the intensity of the driving current increases, heat is generated, and the magnetic field is reduced. In order to solve this problem, the coil temperature is kept constant by using a cooling system, thus generating a stable magnetic field. Using a three-axis orthogonal robot system, as well as magnetic simulations, we observed and analyzed the 3D magnetic field distribution in the working space during pinch motions. A Gauss meter probe was installed in the three-axis orthogonal robotic system.

Furthermore, the developed system includes a monitoring system for the driving conditions and finger motion analysis; using LabView software, the developed motion analysis program monitors the driving current, finger motion, and finger angles during flexion and extension in real time. Finger motion analysis is performed by image processing using a camera and a marker attached to the tip of the finger. The images collected through the camera are binarized to shorten the computation time. The position of the marker was tracked using the pattern matching method, and the angle information of the arrow was collected using the corner detection method. The collected current, time, and angle data are reorganized as current-angle and time-angle graphs, respectively, and displayed on the GUI in real time.

To verify the occurrence of pinch motions due to magnetic force and torque, we fabricated two passive-type robotic fingers. In this configuration, a force of up to 2.5 N was generated during the pinch motion. The proposed system and device are not applicable if fingers are hardened. The system is an apparatus for actively assisting pinch motion by applying a person who can actuate the fingers. Therefore, the generated force up to 2.5 N is suitable for helping pinch motion. To increase the driving force, if the strength of the driving current is increased or a larger magnet generates the magnetic moment of the finger device, the driving force could be sufficiently improved.

In general, motion trajectory is analyzed by time-dependent variables. However, we considered the motion trajectory according to changes in time and driving current (hysteresis) for motion analysis. Through this study, we were able to verify the applicability of a simpler, easier to wear, and improved hand rehabilitation mechanism using a magnetic wireless actuator and control method. In the proposed control system, the hand position must be fixed for rehabilitation training because a single driving coil is used. Therefore, we are developing a three-axis coil-based control system that enables more convenient rehabilitation training. By sensing the hand position, the control system under development would automatically change the driving coil or the current phase to generate a magnetic field suitable for flexion and extension in any given position.

The developed system can programmatically set parameters such as operating time and driving speed in GUI. Therefore, according to the user’s conditions, it can be set by the professional knowledge of the trainer. In addition, the user can change the setting at any time when using it directly. In particular, an emergency button is prepared for emergencies. Therefore, we can avoid problems with safety and fatigue that may arise during treatment or training. In addition, the developed device operates in a local low-frequency magnetic field under 1 Hz and is not affected by the magnetic field at a distance of 30 cm or more from the driving coil. In particular, substantial magnetic objects must be removed from the hand for accurate finger movement except for the driving device.

## Data Availability

Data can be requested from the corresponding authors.

## Supplementary Materials

The following are available online at https://www.mdpi.com/article/10.3390/s21186216/s1, Video S1: Actively assistive pinch motion using the proposed method. Video S2: Automatic magnetic field measurement. Video S3: Pinch motion using passive type mechanical finger. Video S4: Pinch motion using passive type mechanical finger according to changes in the thickness of the rubber-ped. Video S5: Pinch motions by fingers.
